# Supplementary material for: Structural insight into the mechanism of energy transfer in cyanobacterial phycobilisomes
Source: Nat Commun. 2021 Sep 17;12:5497. doi: 10.1038/s41467-021-25813-y (PMC8448738; doi:10.1038/s41467-021-25813-y)
Supplement: Supplementary file 1 — Supplementary Information [file 41467_2021_25813_MOESM1_ESM.pdf]

Structural Insight into the Mechanism of Energy Transfer in Cyanobacterial Phycobilisomes

Lvqin Zheng[1], Zhenggao Zheng[2,3], Xiying Li[2], Guopeng Wang[1], Kun Zhang[2], Peijun Wei[2], Jindong Zhao[2,4,*] & Ning Gao[1,*]

[1]State Key Laboratory of Membranes and Membrane Engineering, Peking-Tsinghua Center for Life Sciences, School of Life Sciences, Peking University, Beijing 100871, China

[2]State Key Laboratory of Protein and Plant Genetic Engineering, School of Life Sciences, Peking University, Beijing 100871, China

[3]College of Life Science, Qingdao University, Qingdao 266071, China

[4]Key Laboratory of Phycology of CAS, Institute of Hydrobiology, Chinese Academy of Sciences, Wuhan, Hubei 430072, China

These authors contributed equally to this work： Lvqin Zheng, Zhenggao Zheng, Xiying Li

*Correspondence to: Jindong Zhao (Email: jzhao@pku.edu.cn) and Ning gao (Email: gaon@pku.edu.cn)

Supplementary Figures

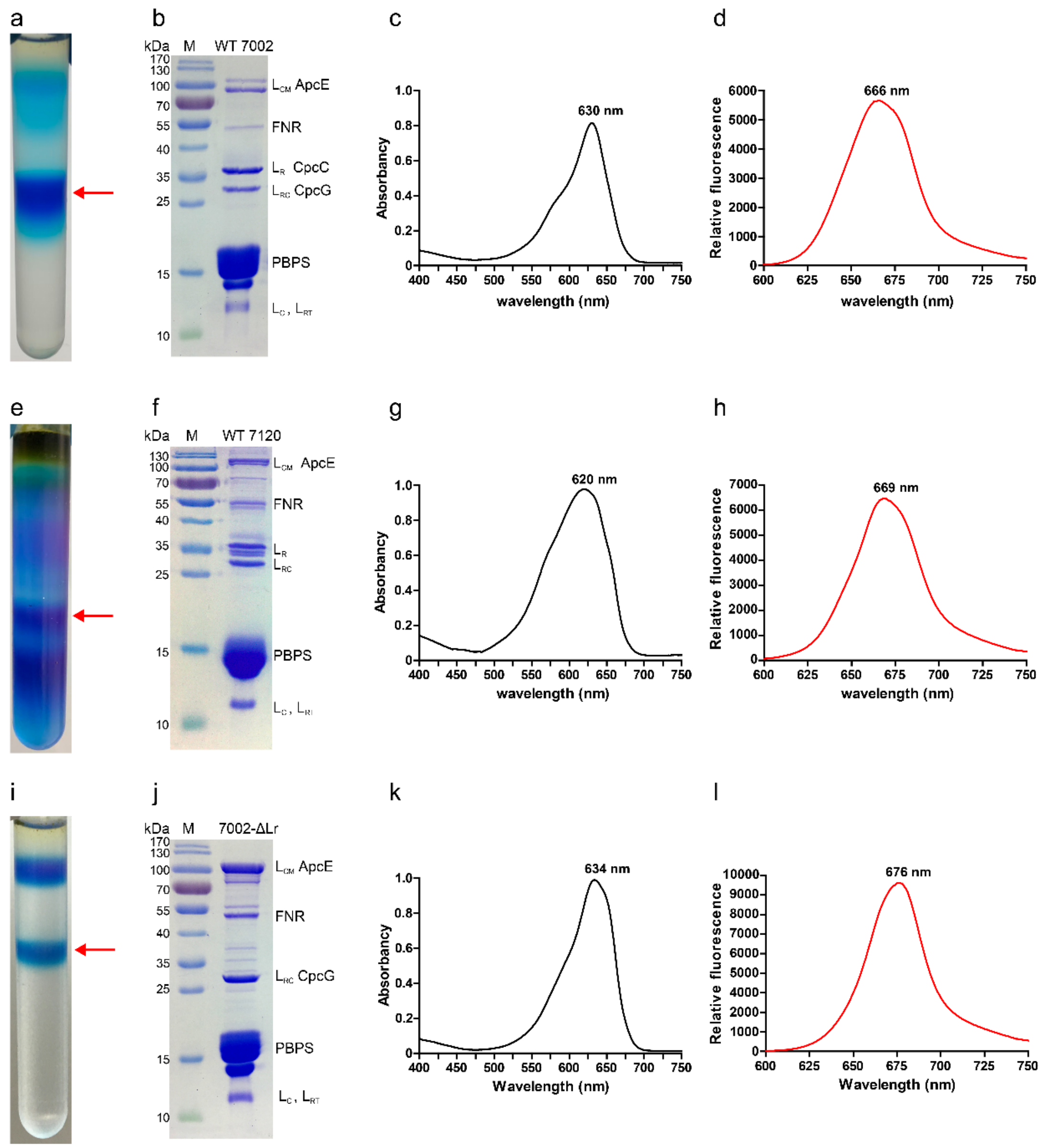

Supplementary Fig. 1 : Preparation and characterization of the PBS from *Synechococcus* 7002, *Anabaena* 7120 and *Anabaena* 7002-△Lr.

a Isolation of phycobilisomes from *Synechococcus* 7002 using sucrose density gradient centrifugation. The samples of the band with red arrow pointing were used for cryo-EM single particle analysis in this study.

b SDS-PAGE analysis of protein components of the PBS from *Synechococcus* 7002. The gel was stained with Coomassie brilliant blue. Experiments were repeated more than three times with similar results.

c Absorption spectrum of the PBS from *Synechococcus* 7002.

d Fluorescence emission spectra of the PBS from *Synechococcus* 7002, excited by 590 nm at room temperature.

e-h Same as a-d, for the PBS from Anabaena 7120.

i-l Same as a-d, for the PBS from Anabaena 7002-△Lr.

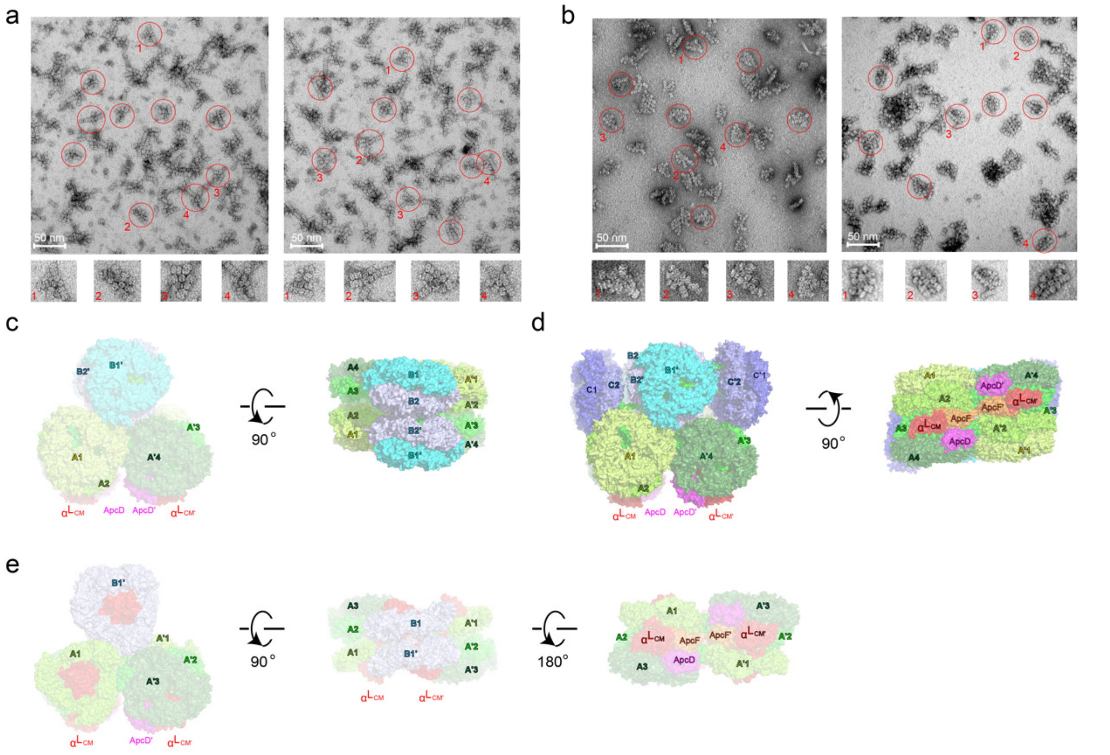

Supplementary Fig. 2 : Negative staining EM of PBS complexes and the structures of the PBS cores.

a Negative staining EM of the PBS from *Synechococcus* 7002. The PBS particles showing different lengths for the rods are indicated by red circles and highlighted in the bottom panels. Experiments were repeated more than three times with similar results.

b Negative staining of PBS from *Anabaena* 7120.

c Side and top views of the arrangement of the core cylinders in *Synechococcus* 7002 PBS.

d Side and bottom views of the arrangement of the core cylinders in *Anabaena* 7120 PBS.

e Different views of the arrangement of the core cylinders in red algal PBS (PDB 6KGX).

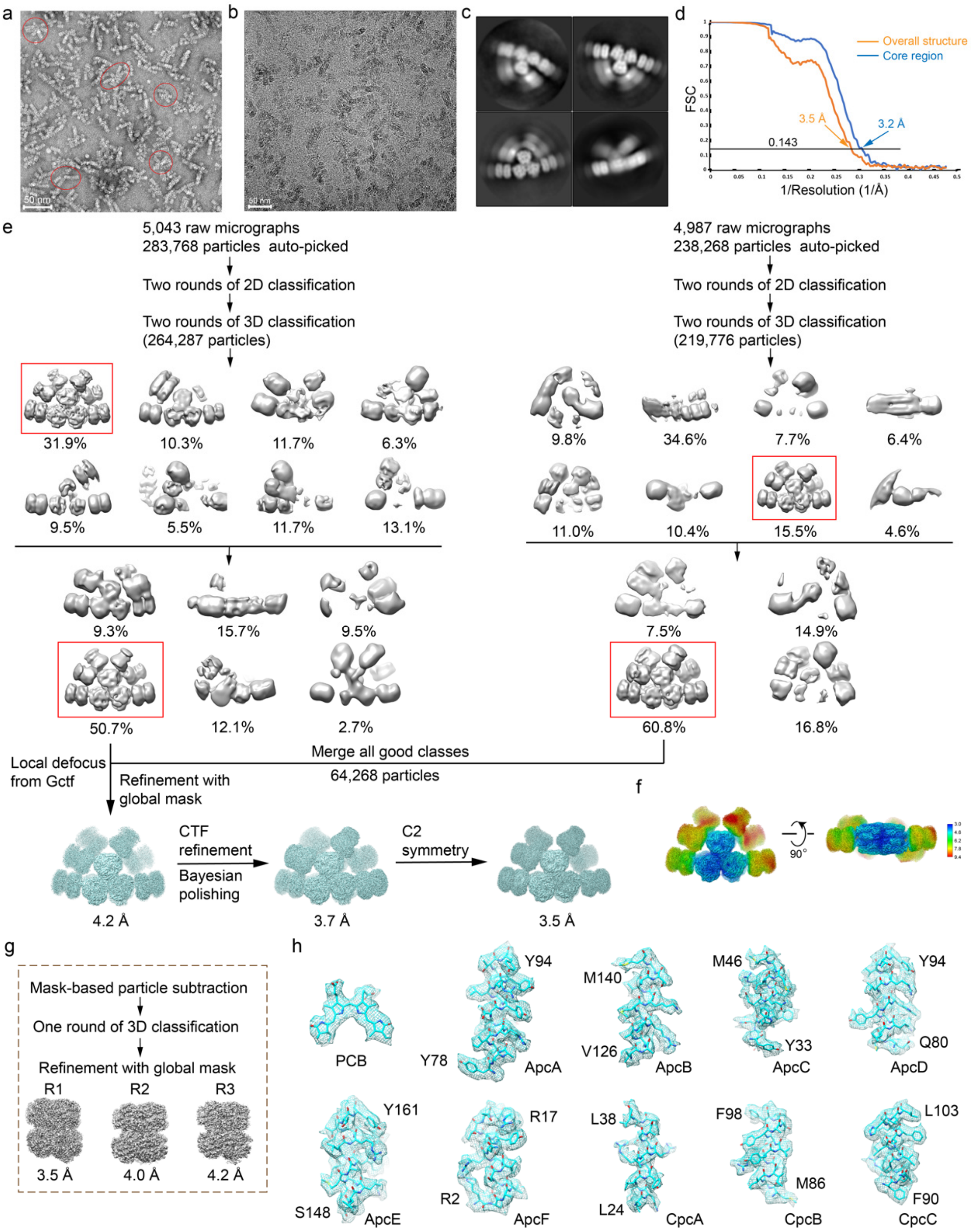

Supplementary Fig. 3 : Image processing of PBS particles from *Synechococcus* 7002.

a Negative staining EM of PBS particles.

b A representative raw cryo-EM image.

c Representative 2D class averages of the PBS particles from *Synechococcus* 7002, showing that peripheral rods are heterogenous in both composition and conformation.

d Gold-standard Fourier shell correction (FSC) of the final cryo-EM maps.

e Image processing workflow.

f Local resolution estimation of the final cryo-EM map.

g Additional image processing procedures for the rod regions.

h Local density of representative PCB and PBS subunits in *Synechococcus* 7002 PBS.

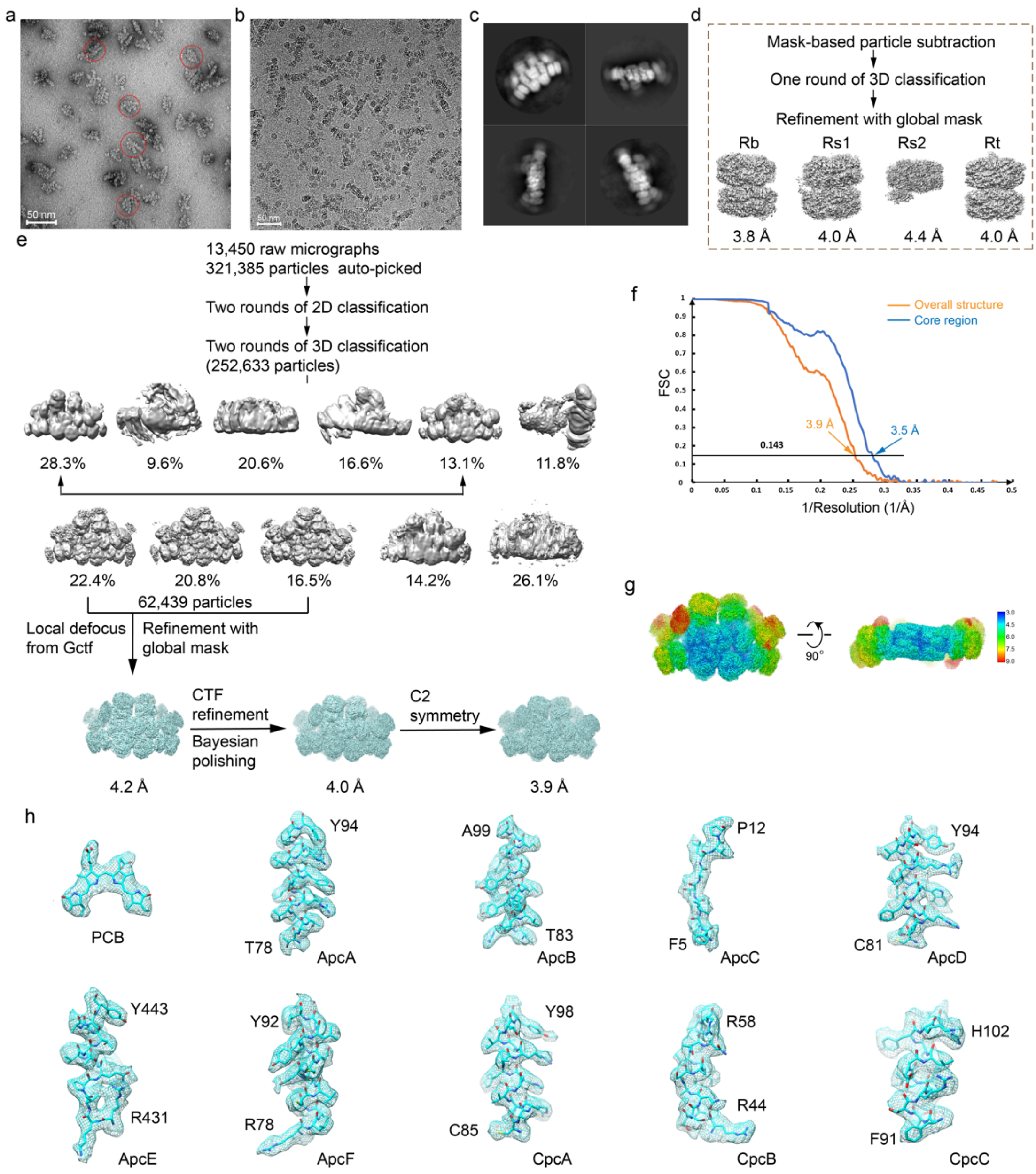

Supplementary Fig. 4 : Image processing of PBS from *Anabaena* 7120.

a Negative staining EM of PBS particles.

b A representative raw cryo-EM image.

c Representative 2D class averages of the PBS particles from *Anabaena* 7120 PBS.

d Additional image processing procedures for the rod regions.

e Image processing workflow.

f Gold-standard Fourier shell correction (FSC) of the final cryo-EM maps.

g Local resolution estimation of the final cryo-EM map.

h Local density of representative PCB and PBS subunits of *Anabaena* 7120 PBS.

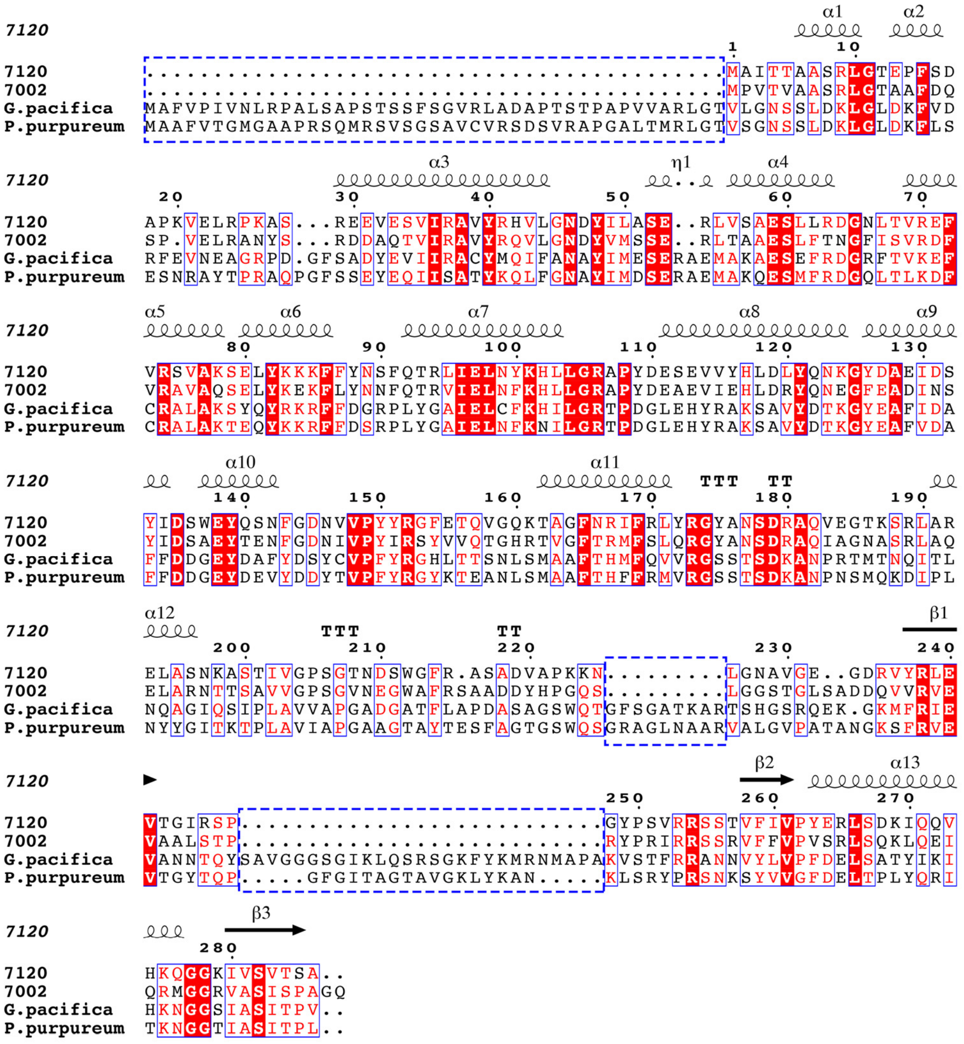

Supplementary Fig. 5 : Sequence alignment of L_R proteins from cyanobacteria and red algae. Alignment of L_R sequences from *Anabaena* 7120, *Synechococcus* 7002, *Griffithsia pacifica* and *Porphyridium purpureum*. The sequence is numbered according to the L_R sequence of *Anabaena* 7120. Three differences between L_R proteins from cyanobacteria and red algae are highlighted in blue dashlined boxes.

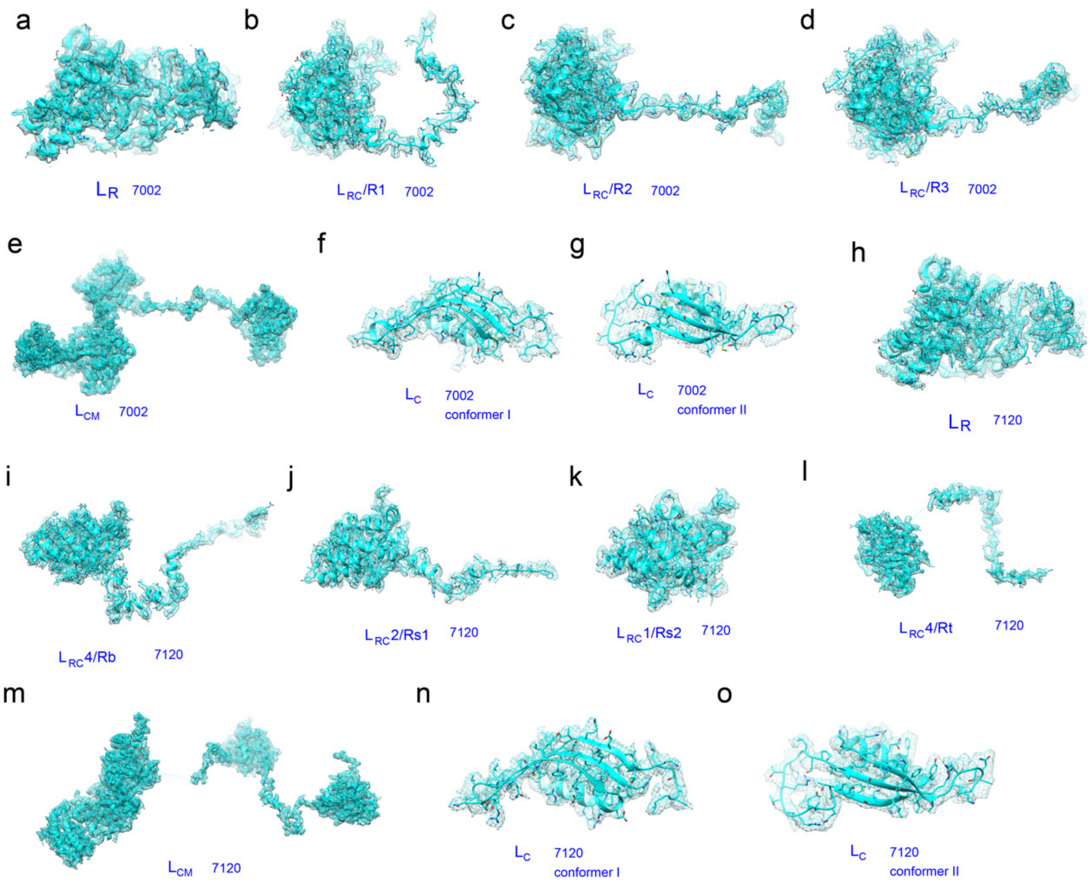

Supplementary Fig. 6: Local densities of PBS subunits.

a Local density of L_R from *Synechococcus* 7002.

b-d Local densities of L_{RC} proteins from *Synechococcus* 7002.

e Local density of L_{CM} from *Synechococcus* 7002.

f-g Local densities of L_C proteins from *Anabaena* 7002.

h Local density of L_R from *Anabaena* 7120.

i-l Local densities of L_{RC} proteins from *Anabaena* 7120.

m Local density of L_{CM} in *Anabaena* 7120.

n-o Local densities of L_C proteins from *Anabaena* 7120.

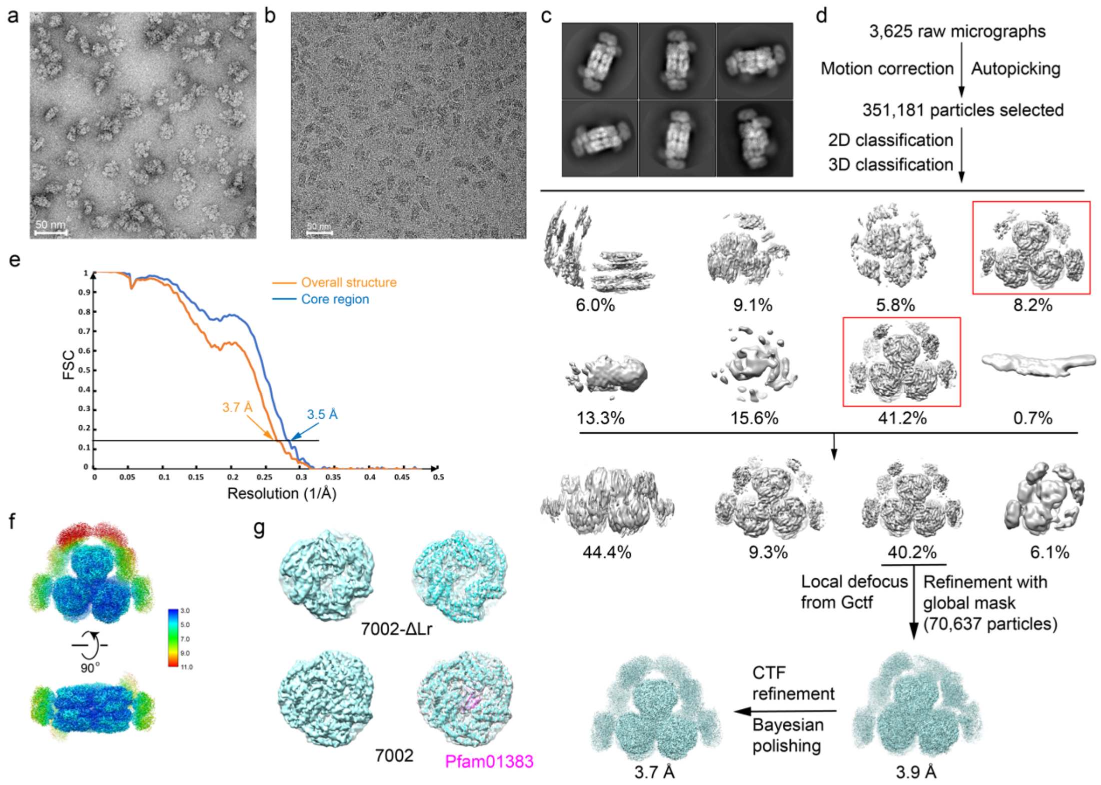

Supplementary Fig. 7 : Image processing of PBS particles from *Synechococcus* 7002-ΔLr.

a Negative staining EM of PBS particles.

b A representative raw cryo-EM image.

c Representative 2D class average of the PBS from 7002-ΔLr.

d Image processing workflow.

e Gold-standard Fourier shell correction (FSC) of the final cryo-EM maps.

f Local resolution estimation of the final cryo-EM map.

g Subunits organization of the rods of the PBS from *Synechococcus* 7002-ΔLr and wild type *Synechococcus* 7002.

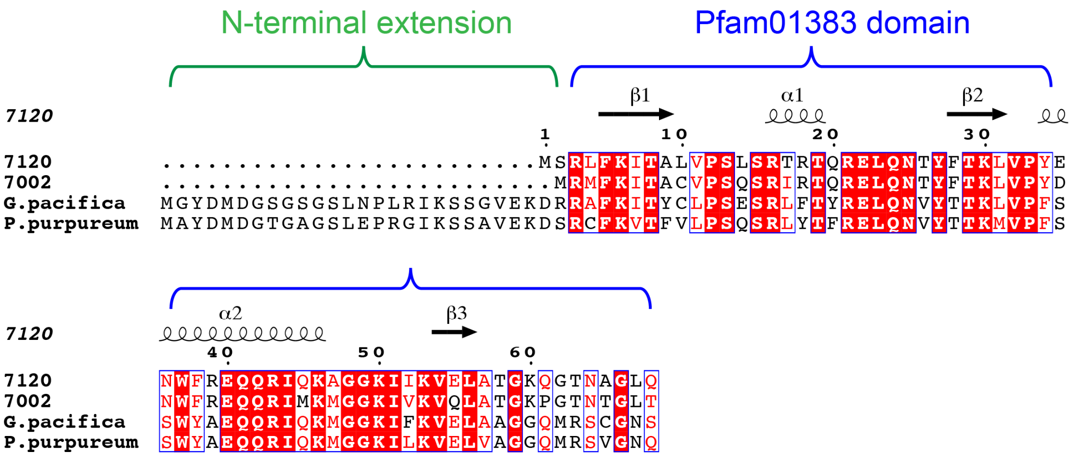

Supplementary Fig. 8 : Sequence alignment of L_C proteins from cyanobacteria and red algae. Alignment of L_C sequences from *Anabaena* 7120, *Synechococcus* 7002, *Griffithsia pacifica* and *Porphyridium purpureum*. The sequence is numbered according to the L_C sequence of *Anabaena* 7120. Compared with the L_C protein in red algae, the L_C proteins in cyanobacteria do not have the N-terminal region.

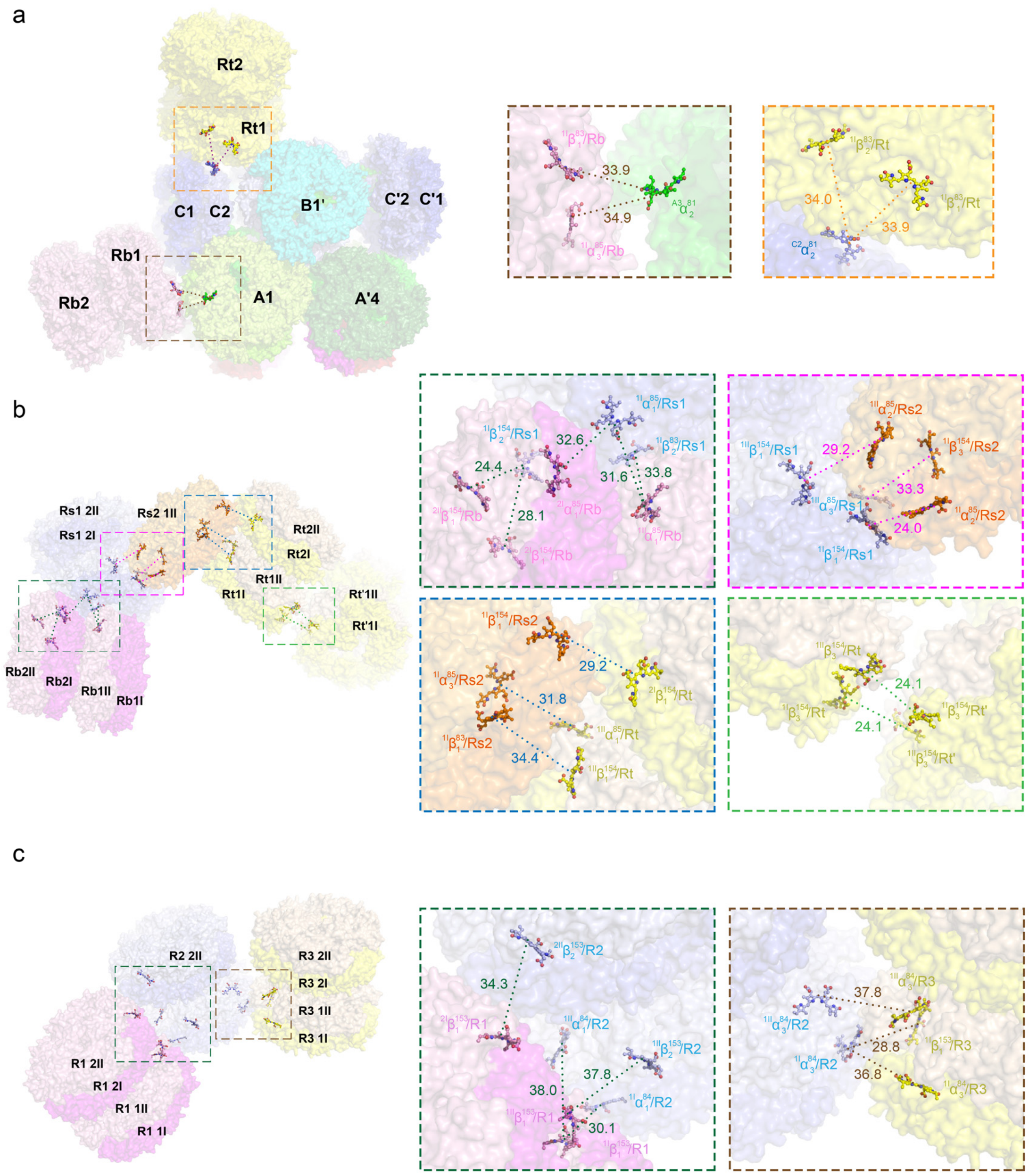

Supplementary Fig. 9 : Plausible energy transfer between rods Rb and Rt the core, and between the rods.

a Plausible energy transfer paths from the bilins on Rb and Rt of *Anabaena* 7120 PBS to the core. The distances (Å) between the closest bilin pairs are indicated, and the bilins are represented as sticks. The coloring scheme is the same as in Fig. 3b.

b Plausible energy transfer paths between the bilins on adjacent rods of *Anabaena* 7120 PBS. Two nearest bilin pairs from adjacent rods are highlighted in the dashlined rectangle boxes.

c Plausible energy transfer paths between the bilins on adjacent rods of *Synechococcus* 7002 PBS.

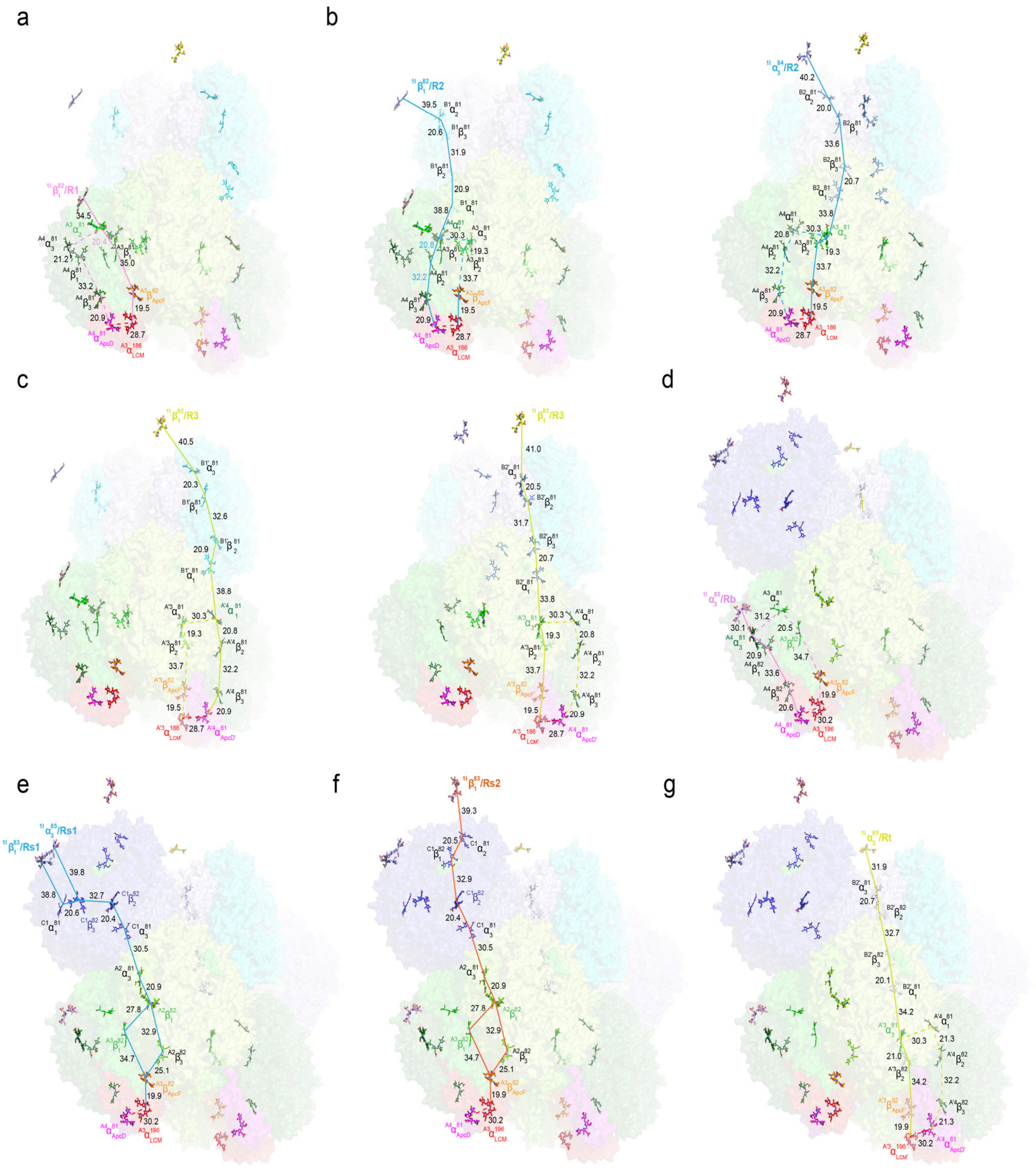

Supplementary Fig. 10 : Possible energy transfer pathways from rods to the core.

a-c Possible energy transfer pathways from rods R1, R2 and R3 to the core in the *Synechococcus* 7002 PBS. The energy transfer routes are colored as Fig. 3a.

d-g Possible energy transfer pathways from rods Rb, Rs1, Rs2 and Rt to the core in the *Anabaena* 7120 PBS. The energy transfer routes are colored as Fig. 3b.

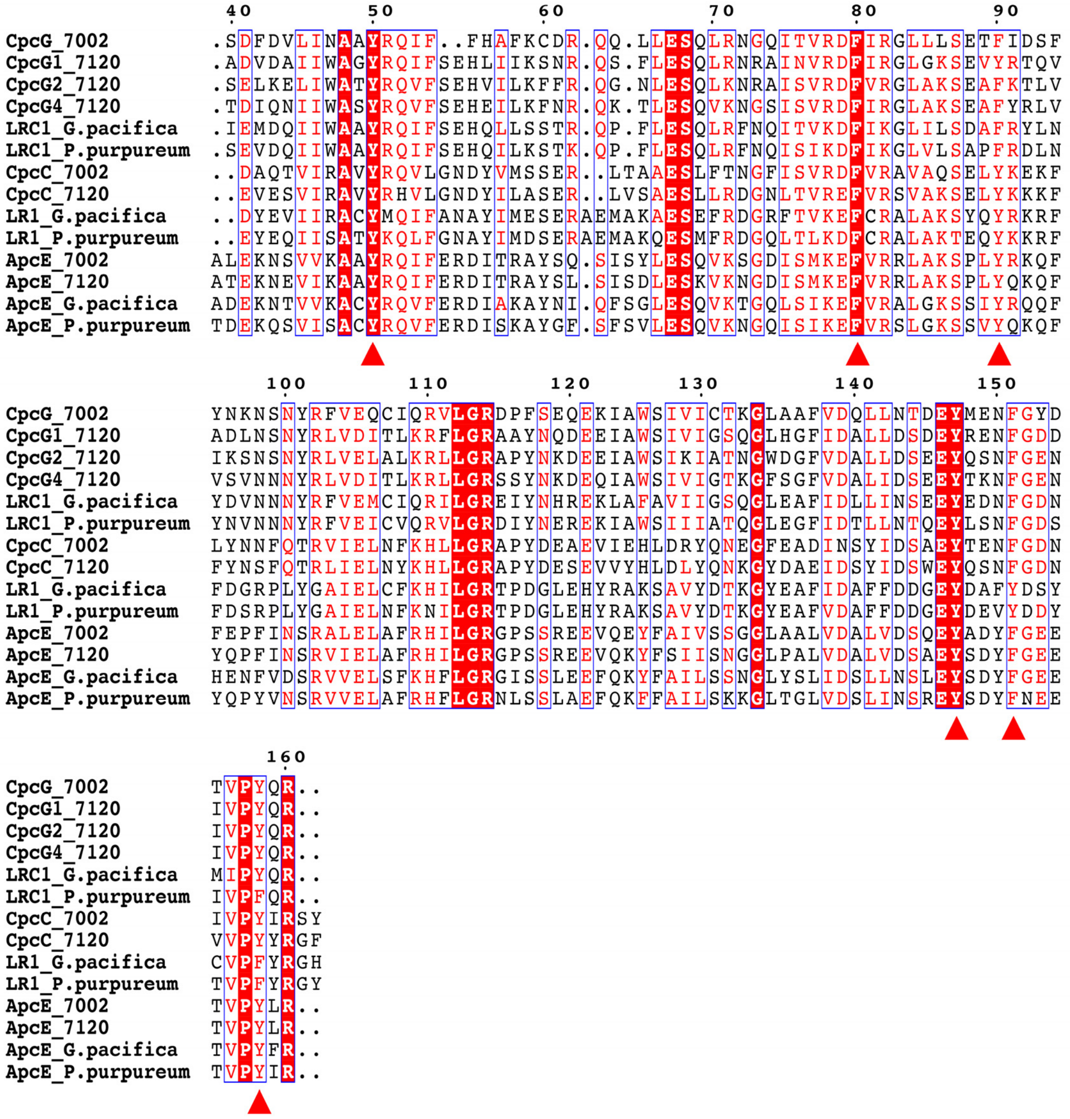

Supplementary Fig. 11 | Sequence alignment of Pfam00427 domain of ApcE, CpcG and CpcC of *Synechococcus* 7002 and *Anabaena* 7120.

Sequence alignment of Pfam00427 domains from *Synechococcus* 7002 and *Anabaena* 7120. The sequence is numbered according to the CpcG sequence of *Synechococcus* 7002. Conserved residues in all Pfam00427 domains are highlighted with red triangles.

Supplementary Tables

Supplementary Table 1 | Sequences of the oligonucleotides used as primers in this study

PapcD L	cccaactacaatattcac
Y88A R	ccgtaggtgaccagacgtagAGCccaaccgtagtcccgcagac
Y88A L	gtctgcgggactacggttggGCTctacgtctggtcacctacgg
apcDem R	aattatagcacgcggtcgacctaggacattgcttgggtaa
apcDem L	ttacccaagcaatgtcctaggtcgaccgcgtgctataatt
em R R	cgcaatcccagaactcaaccctcatgtttgacagcttatc
em R L	gataagctgtcaaacatgagggttgagttctgggattgcg
apcDR R	caaatggctactgtctgc
Y116A R	accggtacaccgagggcgttAGCcatttccttcaccccgatta
Y116A L	taatcggggtgaaggaaatgGCTaacgccctcggtgtaccggt
PapcF L	aagcgcacatcatcaaacac
F60A R	agcagttcagggaccgcttcTGCgagactattggccgcggcct
F60A L	aggccgcggccaatagtctcGCAgaagcggtccctgaactgct
apcFkan R	gcgtgaagcttatcgataccttagagatccacttcgctca
apcFkan L	tgagcgaagtggatctctaaggtatcgataagcttcacgc
kan R R	gttagttgccgtcaactaatcttggtcggtcatttcgaac
kan R L	gttcgaaatgaccgaccaagattagttgacggcaactaac
apcFR R	ctcattgggcacatgacagg
F79A R	atatcccgcaagcaagcagaTGCgcgacgggtcgtataggcat
F79A L	atgcctatacgacccgtcgcGCAtctgcttgcttgcgggatat
F6079A R	ccgcaagcaagcagaTGCgcgacgggtcgtataggcattgcccccagctaagagcagttc agggaccgcttcTGCgagactattggccgc
F6079A L	gcggccaatagtctcGCAgaagcggtccctgaactgctcttagctgggggcaatgcctatac gacccgtcgcGCAtctgcttgcttgcgg
R77K R	cgcaagcaagcagaaaagcgCTTggtcgtataggcattgccc
R77K L	gggcaatgcctatacgaccAAGcgcttttctgcttgcttgcg
R77A R	cgcaagcaagcagaaaagcgCGCggtcgtataggcattgccc
R77A L	gggcaatgcctatacgaccGCGcgcttttctgcttgcttgcg

Supplementary Table 2. Cryo-EM data collection, refinement and validation statistics

	7002 (EMDB-31373) (PDB 7EXT)	7120 (EMDB-31381) (PDB 7EYD)	7002ΔLr (EMDB-31483)
**Data collection and processing**			
Magnification	130,000	130,000	130,000
Voltage (kV)	300	300	300
Electron exposure (e–/Å^2)	64	64	64
Defocus range (μm)	-1.1 to -1.6	-1.1 to -1.6	-1.1 to -1.6
Pixel size (Å)	1.055	1.055	1.052
Symmetry imposed	C2	C2	C1
Initial particle images (no.)	522,036	321,385	315,181
Final particle images (no.)	64,268	62,439	106,308
Map resolution (Å)	3.5	3.9	3.5
FSC threshold	0.143	0.143	0.143
Map resolution range (Å)	3.0-9.4	3.0-11.1	3.0-11.0
**Refinement**			
Initial model used (PDB code)	6KGX	6KGX	
Model resolution (Å)	3.5	3.9	3.5
FSC threshold	0.143	0.143	0.143
Map sharpening *B* factor (Å^2)	-31.877	-103.081	-53.252
Model composition			
Non-hydrogen atoms	322,322	389,750	
Protein residues	40,815	49,454	
Ligands	288	348	
B factors (Å^2)			
Protein	136.72	61.57	
Ligand	146.75	53.43	
R.m.s. deviations			
Bond lengths (Å)	0.008	0.009	
Bond angles (°)	1.101	1.234	
Validation			
MolProbity score	2.19	2.34	
Clashscore	7.71	7.77	
Poor rotamers (%)	0.76	0.02	
Ramachandran plot			
Favored (%)	97.18	95.84	
Allowed (%)	2.82	4.14	
Disallowed (%)	0.00	0.02	
