# Supplementary material for: Structural insight into the mechanism of energy transfer in cyanobacterial phycobilisomes
Source: Nat Commun. 2021 Sep 17;12:5497. doi: 10.1038/s41467-021-25813-y (PMC8448738; doi:10.1038/s41467-021-25813-y)
Supplement: Supplementary file 3 — Description of Additional Supplementary Files [file 41467_2021_25813_MOESM3_ESM.pdf]

Description of Additional Supplementary Files

Supplementary Movie 1: The distribution of aromatic residues of the Pfam00427 domain in the REP1 region of ApcE (*Synechococcus* 7002).

Supplementary Movie 2: The distribution of aromatic residues of the Pfam00427 domain in CpcG (Synechococcus 7002).

Supplementary Movie 3: The distribution of aromatic residues of the Pfam00427 domain in CpcC (Synechococcus 7002).
