# Supplementary material for: Structural insight into the mechanism of energy transfer in cyanobacterial phycobilisomes
Source: Nat Commun. 2021 Sep 17;12:5497. doi: 10.1038/s41467-021-25813-y (PMC8448738; doi:10.1038/s41467-021-25813-y)
Supplement: Supplementary file 7 — Reporting summary [file 41467_2021_25813_MOESM7_ESM.pdf]

nature portfolio

Corresponding author(s): Jindong Zhao and Ning Gao

Last updated by author(s): Aug 19, 2021

Reporting Summary

Nature Portfolio wishes to improve the reproducibility of the work that we publish. This form provides structure for consistency and transparency in reporting. For further information on Nature Portfolio policies, see our Editorial Policies and the Editorial Policy Checklist.

Statistics

For all statistical analyses, confirm that the following items are present in the figure legend, table legend, main text, or Methods section.

n/a	Confirmed	
☐	☒	The exact sample size (*n*) for each experimental group/condition, given as a discrete number and unit of measurement
☐	☒	A statement on whether measurements were taken from distinct samples or whether the same sample was measured repeatedly
☒	☐	The statistical test(s) used AND whether they are one- or two-sided<br>*Only common tests should be described solely by name; describe more complex techniques in the Methods section.*
☒	☐	A description of all covariates tested
☒	☐	A description of any assumptions or corrections, such as tests of normality and adjustment for multiple comparisons
☒	☐	A full description of the statistical parameters including central tendency (e.g. means) or other basic estimates (e.g. regression coefficient) AND variation (e.g. standard deviation) or associated estimates of uncertainty (e.g. confidence intervals)
☒	☐	For null hypothesis testing, the test statistic (e.g. *F*, *t*, *r*) with confidence intervals, effect sizes, degrees of freedom and *P* value noted<br>*Give P values as exact values whenever suitable.*
☒	☐	For Bayesian analysis, information on the choice of priors and Markov chain Monte Carlo settings
☒	☐	For hierarchical and complex designs, identification of the appropriate level for tests and full reporting of outcomes
☒	☐	Estimates of effect sizes (e.g. Cohen's *d*, Pearson's *r*), indicating how they were calculated

Our web collection on statistics for biologists contains articles on many of the points above.

Software and code

Policy information about availability of computer code

Data collection	Cryo-EM images were collected using SerialEM 3.8.7 (http://bio3d.colorado.edu/SerialEM/).
Data analysis	Motion correction was done using MotionCor2 (https://emcore.ucsf.edu/ucsf-motioncor2). CTF parameters were estimated by Gctf (https://www.mrc-lmb.cam.ac.uk/kzhang/Gctf/). 2D and 3D classification, 3D refinement were done using Relion 3.1 (https://www3.mrc-lmb.cam.ac.uk/relion/index.php/Main_Page). Local resolution map was computed by ResMap-1.1.4 (https://github.com/akucukelbir/resmap). Model fitting and building were done using UCSF Chimera-1.11.2 (https://www.cgl.ucsf.edu/chimera/) and Coot-0.8.7 (https://www2.mrc-lmb.cam.ac.uk/personal/pemsley/coot/), respectively. Model refinement was done using Phenix-1.14 (https://www.phenix-online.org/). Sequence alignments were done by Clustal W (https://www.ebi.ac.uk/Tools/msa/clustalo/).

For manuscripts utilizing custom algorithms or software that are central to the research but not yet described in published literature, software must be made available to editors and reviewers. We strongly encourage code deposition in a community repository (e.g. GitHub). See the Nature Portfolio guidelines for submitting code & software for further information.

Data

Policy information about availability of data

All manuscripts must include a data availability statement. This statement should provide the following information, where applicable:

- Accession codes, unique identifiers, or web links for publicly available datasets
- A description of any restrictions on data availability
- For clinical datasets or third party data, please ensure that the statement adheres to our policy

Atomic models of the PBS structures from Synechococcus 7002 and Anabaena 7120 have been deposited in the Protein Data Bank (PDB) under accession number 7EXT (Synechococcus 7002) and 7EYD (Anabaena 7120), respectively. The cryo-EM from Synechococcus 7002, Anabaena 7120 and Synechococcus 7002-ΔLr maps

have been deposited in the Election Microscopy Data Bank with the accession code EMD-31373 (Synechococcus 7002), EMD-31381 (Anabaena 7120) and EMD-31483 (Synechococcus 7002-ΔLr), respectively. Cyanobacterial algae Synechococcus 7002 and Anabaena 7120 are available from the corresponding authors on reasonable request.

Field-specific reporting

Please select the one below that is the best fit for your research. If you are not sure, read the appropriate sections before making your selection.

- [x] Life sciences
- [ ] Behavioural & social sciences
- [ ] Ecological, evolutionary & environmental sciences

For a reference copy of the document with all sections, see nature.com/documents/nr-reporting-summary-flat.pdf

Life sciences study design

All studies must disclose on these points even when the disclosure is negative.

Sample size	No statistic method was used to predetermine the sample size. The number of particles used in structural determination was not pre-determined. The cyanobacterial algae were sufficient for the EM and biochemical analyses. The cryo image are also sufficient as many independently recorded images are acquired as part of cryo-EM data collection.
Data exclusions	Regarding the cryo-EM raw micrograph screening, exclusion was done based on the quality of the images and the presence of ice contamination. Regarding the particle selection, 2D and 3D classification were used and criterion is based on the quality of resulting 2D class average and 3D maps.
Replication	Multiple rounds of structural refinement have been performed and all resulted in same density maps. Every experiment reported was done at least three times with consistent results.
Randomization	No randomization was used other than those embedded in the image processing softwares.
Blinding	No blinding in structural and functional data analysis.

Reporting for specific materials, systems and methods

We require information from authors about some types of materials, experimental systems and methods used in many studies. Here, indicate whether each material, system or method listed is relevant to your study. If you are not sure if a list item applies to your research, read the appropriate section before selecting a response.

Materials & experimental systems

n/a	Involved in the study	
x		Antibodies
x		Eukaryotic cell lines
x		Palaeontology and archaeology
x		Animals and other organisms
x		Human research participants
x		Clinical data
x		Dual use research of concern

Methods

n/a	Involved in the study	
x		ChIP-seq
x		Flow cytometry
x		MRI-based neuroimaging
